# PGCCs Generating Erythrocytes to Form VM Structure Contributes to Tumor Blood Supply

**DOI:** 10.1155/2015/402619

**Published:** 2015-03-25

**Authors:** Shiwu Zhang, Xiaochun Xu, Siwei Zhu, Jun Liu

**Affiliations:** ^1^Department of Pathology, Tianjin Union Medicine Center, Tianjin 300120, China; ^2^Department of Clinical Cancer Prevention, University of Texas, M.D. Anderson Cancer Center, Houston, TX 77030, USA; ^3^Department of Oncology, Nankai University, Tianjin 300121, China; ^4^Department of Medical Imaging, Tianjin Union Medicine Center, Tianjin 300120, China

Over a century ago, it was found that cancer cells often have extra chromosomes; that is, normal human cells contain 46 chromosomes, whereas cancer cells contain abnormal numbers of chromosomes with cell-to-cell variability. Polyploid giant cancer cells (PGCCs) refer to a special subpopulation of cancer cells with giant and multinuclei and contribute to solid tumor heterogeneity. PGCCs differ from normal cells and even other cancer cells in cell size, morphology, proliferation pattern, expression of cell differentiation markers, and chromosome numbers and contribute to tumor formation and chemoradioresistance. The shape of PGCC nuclei is usually irregular and the size is at least three to five times larger than those of regular diploid cancer cells. PGCCs are the key contributor to the heterogeneity of human solid cancers and chromosome structural abnormalities, such as inversions, deletions, duplications, and translocations.

Mechanistically, PGCCs could be formed through end reduplication or cell fusion, reverting to regular cancer cells through splitting, budding, or burst-like mechanisms. PGCCs are divided asymmetrically and cycled slowly to form a dynamic population. However, these giant cells can also revert to regular-sized cancer cells through a reductive division, named as depolyploidization. Asymmetric cell division of giant cancer cells by meiosis-like depolyploidization had been previously proposed to explain the unexpected life cycle of these cells. In this special issue, D. Zhang et al. reported the asymmetric cell division in polyploid giant cancer cells and low eukaryotic cells and revealed the similarities in the budding process between yeast and PGCCs. This mechanism of PGCCs initialed the daughter cell generation which has also been reported in the normal growth of skeletal muscle and osteoclasts and in cells infected by virus or in vitro cell culture. Moreover, PGCCs were able to express certain normal and cancer stem cell markers and differentiate into the adipose tissue, cartilage, and bone. Single PGCC was able to form cancer spheroids in vitro and generate tumor xenograft in immunodeficient mice, indicating that these PGCCs had remarkable biologic features of cancer stem cells.

Furthermore, PGCCs are able to generate erythrocytes in vitro and in vivo besides their cancer stem cell properties. The difference of erythrocytes generated by bone marrow and PGCCs is the different forms of hemoglobin (see below). In human body, erythrocytes are produced in the bone marrow with a process known as hematopoiesis. The bone marrow stroma contains mesenchymal stem cells (MSCs) and hematopoietic stem cells (HSCs), which give rise to erythrocytes, leukocytes, and platelets. In adults, bone marrow is generally considered the main source of erythrocytes. However, PGCCs have an ability to generate erythrocytes in vitro and in vivo.

During cancer development, tumor cells undergo avascular growth. However, after a tumor mass reached a certain size, vasculogenic mimicry (VM) will connect with endothelium dependent vessels to obtain sufficient blood and oxygen supply to support further growth of tumor cells and support tumor invasion and metastasis. Accumulating evidence has demonstrated that different types of cancer utilize VM to form a blood supply network to support their growth, invasion, and metastasis and, clinically, such a tumor is usually associated with poor prognosis. However, the source of erythrocytes in VM remains unclear. PGCCs can be induced by treatment of cancer cells with cobalt chloride (a hypoxia mimic) in vitro and hypoxia will increase self-renewal of cancer stem cells and promote the stem cell-like phenotype besides induction of PGCCs formation. Moreover, hypoxia also promotes the formation of vasculogenic mimicry (VM). B. Sun et al. showed that hypoxia inducible factor-1*α* plays an important role in the VM formation, while L. Zhang et al. provided the evidence that erythroid cells were localized in the cytoplasm of or around the PGCCs in serous ovarian carcinoma tissues and cancer cells in the VM structures and that these erythroid cells expressed hemoglobin-*β*/*γ*/*ε*/*δ* and hemoglobin-*ζ* detected by immunostaining. Thus, these VM structures can be formed by PGCCs or other cancer cells and their newly generated fetal erythrocytes with high O_2_ binding affinity.

In addition, in this special issue, W. Wang et al. and L. Yao et al. demonstrated that epithelial-mesenchymal transition and Wnt signaling pathway could regulate the VM formation. Thus, elucidation of the molecular mechanisms of PGCC and VM formation could provide a novel insight into research in embryology, stem cells, and tumorigenesis. Identification of the PGCCs and tumor-derived erythrocytes could be a survival mechanism in hypoxia and targeting of PGCCs might be further developed as a potential therapeutic strategy for human cancers. Research focus on VM-targeted therapies could include dendritic cell vaccine and cytokine-induced killer cell therapy to conquer the recurrence and metastasis of aggressive cancers.

*Shiwu Zhang* *Xiaochun Xu* *Siwei Zhu* *Jun Liu*

